# The effect of citric acid on mineralisation and vascular endothelial growth factor secretion from apical papilla stem cells

**DOI:** 10.2340/aos.v83.42026

**Published:** 2024-10-01

**Authors:** Krasimir Hristov, Nikolay Ishkitiev, Marina Miteva, Violeta Dimitrova, Ralitsa Gigova, Nataliya Gateva, Liliya Angelova

**Affiliations:** a Department of Pediatric Dentistry, Faculty of Dental Medicine, Medical University of Sofia, Sofia, Bulgaria; b Department of Chemistry and Biochemistry, Medical Faculty, Medical University of Sofia, Sofia, Bulgaria; c Department of Dental Public Health, Faculty of Dental Medicine, Medical University of Sofia, Sofia, Bulgaria

**Keywords:** Citric acid, VEGF, regenerative endodontics, stem cells from apical papilla, immature teeth

## Abstract

**Objective:**

To investigate the influence of citric acid on the osteogenic and angiogenic potential of stem cells from apical papillae (SCAPs).

**Materials and methods:**

Stem cells from apical papillae were isolated from freshly extracted third permanent molars. These cells were treated with 20 and 100 μM citric acid. Alizarin red staining was used to evaluate mineral deposition. The secreted levels of vascular endothelial growth factor (VEGF) were assessed by ELISA on days 18, 24 and 28. Immunofluorescence analysis was performed to assess the expression of surface markers after exposure to 20 and 100 μM citric acid.

**Results:**

Different mineralisation patterns were observed. Supplemented with citric acid, media showed more diffuse and less dense crystals. On day 18, most VEGF was secreted from the cells with no added citric acid. On day 24, there was a significant increase (*p* < 0.05) in the levels of VEGF secreted from cells treated with 20 μM citric acid. On day 28, cells from the control group did not secrete VEGF. There was a reduction in the levels of VEGF secreted by cells treated with 20 μM citric acid and a significant increase in the cells exposed to 100 μM citric acid (*p* < 0.05).

**Conclusion:**

Citric acid can promote the differentiation of SCAPs and angiogenesis.

## Introduction

Regenerative endodontic treatment (RET) is becoming increasingly popular in managing apical periodontitis in immature permanent teeth [1]. This procedure permits the lengthening and thickening of the walls of the root canal, construction of the apex, and in some cases, may even promote the recovery of tooth sensitivity [2]. The most complex aspect of this treatment is the formation of a new pulp-dentin complex, which requires the predictable induction of angiogenesis and neurogenesis [3]. As with conventional endodontic treatments, thorough disinfection of the root canals and removal of the smear layer are of utmost importance for a successful outcome [4]. Removal of the smear layer increases penetration of the irrigant and subsequently enhances root canal disinfection and the release of growth factors [5]. In regenerative endodontics, there are several key considerations in addition to the antibacterial effect of irrigants: low cytotoxicity and the ability to release growth factors included in the dentin [6, 7]. Growth factors from the dentin, which play a key role as biological inducers, are reactivated and released during its demineralisation under the influence of acidic products [8]. In endodontic treatment, this is achieved through using organic acids or chelators as irrigants [9, 10]. Ethylenediaminetetraacetic acid (EDTA) and citric acid are the most commonly used solutions to achieve this goal in clinical practice [11]; these products favour cell adhesion and have no adverse effects on cell proliferation [12, 13]. Stimulation of the expression of growth factors by undifferentiated stem cells is also essential [14]. Therefore, the aim of the present study was to investigate the influence of citric acid on the osteogenic and angiogenic potential of stem cells from apical papillae (SCAPs).

## Materials and methods

### Isolation of stem cells from apical papillae

Firstly, we isolated SCAPs from freshly extracted tooth germs of the permanent third molars. The growth of the SCAPs was maintained in cell culture. The apical papillae were carefully removed from the tooth’s root and then stored and incubated in an enzymatic digestion solution containing 3 mg/mL of collagenase (Sigma-Aldrich, St. Louis, MO, USA) and 4 mg/mL of dispase at 37°C for 1 hour. A single-cell suspension was obtained by passing the cells through a sterile 70 µm strainer (Falcon, Corning, NY, USA) and seeding the cells into culture dishes containing Dulbecco’s Modified Eagle Medium (DMEM) (Invitrogen, Eugene, OR, USA). The culture medium was supplemented with 10% foetal bovine serum, 100 U/mL of penicillin, and 100 µg/mL of streptomycin (Invitrogen). Cells between the third and fifth passages were used for subsequent experiments.

### Immunofluorescence assays

The stem cell properties of SCAPs were demonstrated by assessing the expression levels of several cell surface markers by immunocytochemistry, and the use of several primary antibodies: STRO-1 (Thermo Fisher Scientific, Waltham, MA, USA), CD24, CD31, CD13, and CD146. Except for STRO-1, all antibodies were produced in mice by Sigma-Aldrich (Sigma-Aldrich, St. Louis, Missouri, United States). Cells were washed with phosphate-buffered saline (PBS) (Biolegend, San Diego, CA, USA). Thereafter, non-specific binding sites were blocked by incubation in 2% bovine serum albumin (BSA) in PBS, re-washing with PBS, incubating with the primary antibody for 1 hour at room temperature, washing with PBS, and labelling with an anti-mouse secondary antibody for 1 hour in the dark (Sigma-Aldrich).

### Mineralisation assessment

Cells were cultured for 14 days in four different types of media. For the control group, cells were cultured in DMEM, supplemented with 10% foetal bovine serum (Sigma -Aldrich), 100 U/mL of penicillin, 100 µg/mL of streptomycin, and 10 ng/mL of TGFβ (RnD Systems, Minneapolis, MN, USA). Cells in mineralisation group 1 (MM1) were cultured in DMEM supplemented with 50 μg/mL of ascorbic acid (Serva Electrophoresis GmbH, Heidelberg, Germany), 10 nM dexamethasone (Wako Pure Chemicals, Osaka, Japan), and 10 mM β-glycerophosphate (Sigma-Aldrich). Cells in mineralisation group 2 (MM2) were cultured in DMEM supplemented with 50 μg/mL of ascorbic acid, 10 nM dexamethasone, 10 mM β-glycerophosphate, and 20 μM of citric acid. Cells in mineralisation group 3 (MM3) were cultured in DMEM supplemented with 50 μg/mL of ascorbic acid, 10 nM dexamethasone, 10 mM β-glycerophosphate, and 100 μM of citric acid.

Alizarin red staining was used to evaluate mineral deposition. For this purpose, the culture medium was aspirated from the wells of culture plates and the cells were washed three times with PBS. This was followed by their fixation with 10% formalin for 1 hour at room temperature. The solution was then aspirated, and the cells were washed thrice with deionised water. At room temperature and dark conditions, 1 mL of fresh Alizarin red solution (Applichem GmbH, Darmstadt, Germany) was added to each well and incubated for 45 minutes. Subsequently, the solution was removed and the cells were washed thrice with deionised water. Finally, 1 mL of PBS was added to each well and the cells were observed using a phase-contrast light microscope (Leica Camera, Wetzlar, Germany).

### Vascular endothelial growth factor expression during cell culture

The levels of vascular endothelial growth factor (VEGF) secreted by cultured cells were determined in three types of culture media: (1) DMEM supplemented with 10% foetal bovine serum, 100 U/mL of penicillin, and 100 µg/mL of streptomycin; (2) DMEM supplemented with 10% foetal bovine serum, 100 U/mL of penicillin, 100 µg/mL of streptomycin, and 20 µM citric acid; and (3) DMEM supplemented with 10% foetal bovine serum, 100 U/mL of penicillin, 100 µg/mL of streptomycin, and 100 µM citric acid. The supernatants from each well were collected on days 18, 24, and 28, and the levels of secreted VEGF were assayed by ELISA (Human VEGF-B ELISA Kit, Sigma Aldrich). Immunofluorescence analysis was also performed to investigate the expression of stem cells and differentiation markers.

### Statistical analysis

Each experiment was carried out three times. Data were collated and analysed by Statistical Package for the Social Sciences (SPSS) version 19.0 software (IBM Corp., Armonk, NY, USA). Shapiro-Wilk test was used to determine whether the data set was normally distributed (*p* > 0.05). Data were compared by analysis of variance (ANOVA), and differences were considered significant at *p* < 0.05.

## Results

**Figure 1:**
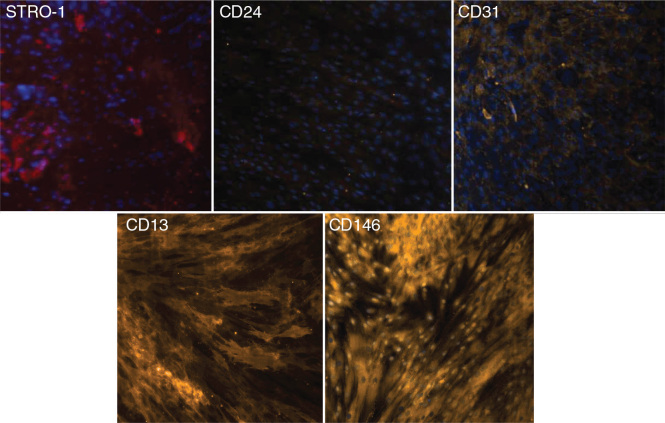
Immunofluorescence study of the expression of cell surface markers (STRO-1, CD24, CD31, CD13, and CD146) in apical papilla stem cells.

Immunofluorescence was used to qualitatively investigate the ability of cells isolated from apical papillae to express surface markers. The resulting images are presented in Figure 1. These images confirm the presence of stem cells in the apical papillae because of the expression of cell markers that are typical for undifferentiated cells. Cells did not express individual markers in a homogenous manner. These results confirm the heterogeneous nature of the cell cultures.

**Figure 2:**
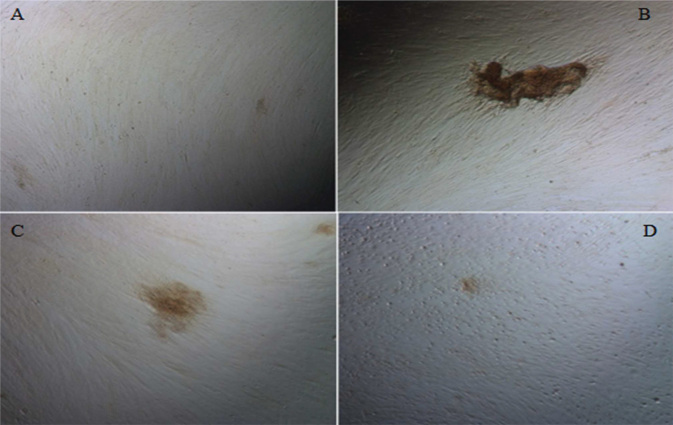
Evaluation of the formation of mineral deposits by SCAPs after exposure to citric acid. (A) Control, (B) Standard mineralisation-inducing medium, (C) Medium supplemented with 20 μM citric acid, and (D) Medium supplemented with 100 μM citric acid. SCAP: stem cells from apical papillae.

Figure 2 shows mineral deposits after the incubation of SCAPs in different osteo-differentiation media, as visualised by Alizarin red staining. Different mineralisation patterns were observed, with cultures with standard osteogenic medium characterised by denser and localised mineral deposits. Cells with different concentrations of citric acid added were more diffuse and less dense. The deposits were non-homogeneous, and their size and density decreased with increasing citric acid concentration.

**Figure 3:**
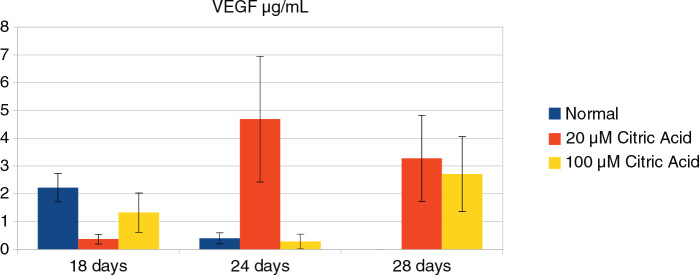
The levels of secreted VEGF in control medium (blue), medium supplemented with 20 μM (red), and 100 μM citric acid (yellow). VEGF: Vascular Endothelial Growth Factor.

Figure 3 shows the levels of VEGF secreted on days 18, 24, and 28. On day 18, the levels of secreted VEGF were the highest from cells cultured in the absence of citric acid; the next highest levels of VEGF were secreted by cells exposed to 100 μM citric acid. There was a significant difference between all individual groups (ANOVA *p* < 0.05). On day 24, there was a significant increase in the levels of secreted VEGF from cells cultured in a medium supplemented with 20 μM citric acid; however, the levels of VEGF secreted by cells in the control group and those exposed to 100 μM citric acid decreased significantly (*p* < 0.05). On day 28, cells from the control group did not secrete VEGF. There was a reduction in the levels of VEGF secreted by cells cultured with 20 μM citric acid and a significant increase in the levels of VEGF secreted by cells exposed to 100 μM, although these levels did not reach those produced by cells exposed to 20 μM citric acid (ANOVA, *p* < 0.05).

**Figure 4:**
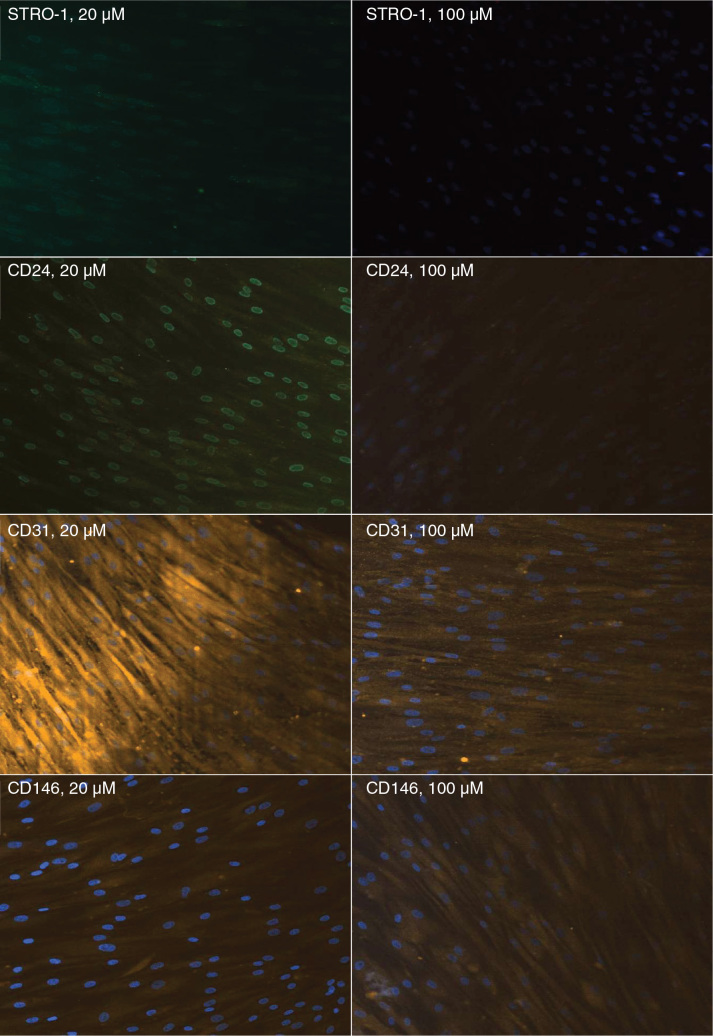
Immunofluorescence analysis of the expression of surface markers by SCAPs after exposure to 20 and 100 μM citric acid. SCAP: stem cells from apical papillae.

Immunofluorescence analysis revealed a weakening of positive signals associated with surface markers when the cells were incubated in a medium containing citric acid; this was most likely because of the initiation of cellular differentiation (Figure 4).

## Discussion

In the present study, we investigated the osteogenic and angiogenic potential of citric acid on SCAPs. Citric acid and its citrate derivatives represent a metabolic factor that is found in abundance in bone and can be used by mesenchymal stem cells to stimulate osteogenesis by regulating certain metabolic pathways [15]. Citric acid is known to have antioxidant and anti-inflammatory properties [16]. In addition, using 10% citric acid as an irrigant has been shown to promote the release of dentin-incorporated growth factors without affecting cell proliferation and adhesion to the root dentin [12]. In some cases, conditioning dentin with citric acid has been shown to promote the release of greater amount growth factors than EDTA [8, 15]. The concentrated release of growth factors can accelerate tissue regeneration by stimulating cell proliferation and the differentiation of stem cells [17, 18].

Of the various surface cell markers, CD24, a pluripotency marker, has been shown to be directly associated with and predominantly expressed in SCAPs, although expression levels are usually low [19]. This marker could not be detected in other types of mesenchymal stem cells, including cells from dental pulp [20, 21]. CD24 is used as an indicator for an undifferentiated cell state; therefore, its presence indicates the stemness of cells. As the level of alkaline phosphatase increases, the expression level of CD24 decreases, thus indicating that cells have begun to leave the undifferentiated state and enter that of the osteoblastic nature/lineage [22]. On the other hand, regardless of differentiation, once cells reach the 10th passage, CD24 expression disappears. Given that CD24 is considered as an identifying marker of SCAPs, this finding suggests that the stem cell properties of SCAPs are reduced with passage [23]. Some of the cells in the present study expressed CD24; we also observed that the signal intensity weakened after incubation in a citric acid medium, probably because of the initiation of differentiation processes (Figures 1 and 4). Our results are consistent with publications in the specialised literature concerning the expression of the CD24 marker by SCAPs [20, 21] but in medium supplemented with citric acid.

The analysis of CD146 expression is vital because of the fact that CD146 is the most commonly used marker to characterise perivascular pluripotent stem cells in connective tissue [24]. As with all markers, the expression of CD146 is not homogeneous and requires fine cell sorting to obtain a pure population [25]. Furthermore, because of variations in their expression, neither CD146 nor STRO-1 can be used alone as specific markers of dental stem cells [26]. In an isolated population of SCAPs, CD146-positive cells predominated over STRO-1-positive cells [27]. STRO-1-positive cells have been shown to exhibit neurogenic characteristics, staining positive for beta-3-tubulin, nestin, and neuron-specific enolase, which are all neuronal stem markers. This supports the claim that these cells are oriented towards specific cell lineages depending on the proportional expression of certain factors [28].

Stem cells from apical papillae have the potential for multi-linear differentiation, extracellular matrix synthesis, and mineralisation. These cells can differentiate into odontoblasts/osteoblasts, adipocytes, chondrocytes, and neurocytes *in vitro* in the presence of established cell line-specific active substances such as growth factors and cytokines [29–31]. This property may be because of the presence of certain factors in heterogeneous cell culture: (1) multipotent stem cells, (2) precursor cells such as odontoblasts/preosteoblasts/preadipocytes, chondroblasts or neural cell precursors, or (3) a combination of all of these factors [32].

The potential of SCAPs to undergo odontoblastic/osteoblastic differentiation was investigated 30 days after induction with an osteogenic medium, where they could form small round Alizarin red–positive nodules which were indicative of calcium deposition *in vitro* [33]. Similar to dental pulp stem cells, SCAPs possess odontogenic potential *in vitro*. These cells express lower levels of dentin sialoprotein (DSP), matrix extracellular phosphoglycoprotein (MEPE), transforming growth factor (TGF)β-II receptor, fibroblast growth factor receptor (FGFR1), FGFR3, and melanoma-associated glycoprotein (CD146/MUC18) when compared to dental pulp stem cells [34]. Upon the transplantation of SCAPs with hydroxylapatite/tricalcium phosphate (HA/TCP) granules into immunocompromised mice, these cells underwent differentiation into odontoblasts, thus regenerating a dentin/pulp-like structure and connective tissue. Furthermore, the co-transplantation of SCAPs and periodontal ligament stem cells with HA/TCP granules resulted in the formation of dentin and periodontal ligament [35]. It was confirmed in the current study that SCAPs possessed the potential for osteogenic/odontogenic differentiation. This was demonstrated by visualising mineral deposits after alizarin red staining (Figure 2). In the medium supplemented with citric acid, these deposits were smaller in size and density, probably because of the lower pH of the environment. On the other hand, citrate supplementation increased the production of collagen and mineralisation, thus influencing the organisation of the mineral matrix [36]. This *in vitro* study proves that irrigation of the root canal with citric acid may promote the process of cell differentiation (Figure 3).

Vascular endothelial growth factor is a vascular endothelial cell mitogenic factor that modulates physiological angiogenesis, vascular permeability, cell migration, proliferation, and vasodilation. In addition, VEGF is a positive regulator of bone development, skeletal growth and fracture healing, and can also stimulate the proliferation and differentiation of osteoblasts [37, 38]. Under appropriate conditions, VEGF can induce the differentiation of stem cells into endothelial cells while modulating tooth development and dentinogenesis [39]. Research has shown that VEGF is synthesised by human pulp cells under physiological conditions and at greater levels in pathological conditions, thus leading to the increased proliferation and expression of alkaline phosphatase [40, 41]. FGF-2, platelet-derived growth factor (PDGF), and TGFβ are released after the application of orthodontic force [42] and act synergistically on pulp angiogenesis both *in vitro* and *in vivo* [43]. Hydrogels loaded with VEGF are known to increase proliferation, alkaline phosphatase activity, mineralisation and osteogenesis in undifferentiated stem cells [44].

Vascular endothelial growth factor induces the differentiation of dental pulp stem cells into endothelial cells. Under osteogenic conditions, treating dental pulp stem cells with VEGF leads to their differentiation into osteoblasts, as demonstrated by Alizarin red staining and an increase in alkaline phosphatase activity [45]. VEGF-modified dental pulp stem cells exhibit increased myelination, and an increased thickness and diameter of axons in nerve tissue [46]. Exposing SCAPs to VEGF is known to induce the expression of factors that are typical of odontoblasts and neuroblasts, thus providing evidence for the critical role of this VEGF in regenerative endodontic procedures [3]. In the present study, adding citric acid to the culture medium over the first 18 days of SCAPs culture did not increase the levels of secreted VEGF when compared to cells cultured in a standard medium (the control group). However, a significant increase in the levels of VEGF were observed after the 24th day in cells exposed to citric acid; 20 µM citric acid had a better effect than 100 µM (Figure 3). Collectively, the results of the study show that the use of citric acid as an irrigant solution in the protocols of regenerative endodontic procedures may have a positive effect on cell proliferation, differentiation, and angiogenesis by increasing the amount of VEGF released.

## Conclusions

Using citric acid as an irrigant in the protocols of regenerative endodontics, in addition to a disinfecting and conditioning effect, stimulates the differentiation of stem cells from the apical papillae. Furthermore, citric acid significantly increases the levels of VEGF after the 28th day of SCAPs culture; this may stimulate angiogenesis in regenerative endodontic procedures and contribute to the successful outcome of the treatment over the long term.
